# Cytogenomic delineation and clinical follow-up of 10 Brazilian patients with Pallister-Killian syndrome

**DOI:** 10.1186/s13039-015-0142-7

**Published:** 2015-06-26

**Authors:** Larissa Sampaio de Athayde Costa, Aline C. Zandona-Teixeira, Marilia M. Montenegro, Alexandre T. Dias, Roberta L. Dutra, Rachel S. Honjo, Debora R. Bertola, Leslie D. Kulikowski, Chong A. Kim

**Affiliations:** Laboratório de Citogenômica, LIM 03, Departamento de Patologia, Faculdade de Medicina da Universidade de São Paulo- HCFMUSP, São Paulo, Brasil; Unidade de Genética, Departamento de Pediatria, Instituto da Criança-HCFMUSP, Universidade de São Paulo, São Paulo, Brasil

**Keywords:** MLPA, Buccal smear, Pallister-Killian syndrome, isochromosome 12p

## Abstract

**Background:**

Pallister-Killian syndrome (PKS) is a sporadic genetic disorder caused by the presence of a tissue-specific mosaicism for isochromosome 12p - i(12) (p10) and is characterized by facial dysmorphism including coarse facies, upslanting palpebral fissures, bitemporal alopecia, pigmentary skin anomalies, developmental delay, hypotonia and seizures. Although typical clinical features of PKS commonly exist, clinicians often do not raise the possibility of this diagnosis.

**Results:**

We reviewed the medical records of 10 patients with confirmed PKS followed in our service (since 1990 to 2015). Age at diagnosis varied from prenatal to 3 years and clinical features were consistent with those described in the literature. In all patients, peripheral blood karyotypes were normal and cytogenomic study was performed in order to confirm the diagnosis. Three of these patients had PKS diagnosis confirmed by buccal smear MLPA.

**Conclusion:**

An early conclusion from our results demonstrated that MLPA on buccal smears is a good and non-invasive method to detect extra copies of 12p and should be considered as the first exam, before a skin biopsy for a fibroblast karyotype is performed.

## Background

Pallister-Killian syndrome (PKS) is a sporadic and rare chromosomal disorder caused by the mosaicism for isochromosome 12p – i(12)(p10). There are just over 200 cases described in the literature [1]. The syndrome is known by several names, such as Pallister mosaic syndrome, Pallister-Killian syndrome, Pallister-Killian-Teschler-Nicola syndrome, tetrasomy 12p and isochromosome 12p [2, 3].

Clinical findings of PKS are distinctive, especially the combination of coarse facies, pigmentary skin anomalies, developmental delay, hypotonia and seizures. The facial appearance includes coarse facies (coarseness becomes more pronounced with age), prominent forehead, hypertelorism, upslanting palpebral fissures with epicanthal folds, small nose with upturned nares, high arched palate, macrostomia, long philtrum, micrognathia, bitemporal alopecia and an extension of the philtral skin into the vermilion border of the upper lip, which is termed “Pallister lip”. Other associated findings include the following: congenital heart defects, diaphragmatic hernia, cryptorchidism, renal malformations, imperforate or anteriorly placed anus [2–4]. Patients have hypotonia, mental and motor retardation from early infancy, which can vary from mild to severe and profound [2, 5–7].

PKS is caused by a tissue-limited mosaicism for supernumerary i(12)(p10) [4, 8]. Although different levels of mosaicism and genetic variation in isochromosome composition have already been described in the literature, there is currently no evidence for genotype-phenotype correlation [3].

A fibroblast karyotype from skin biopsy is commonly used to achieve PKS diagnosis, but other tissues such as lung, tissue from a buccal smear, bone marrow and even amniotic fluid yield higher percentages of mosaicism i(12)(p10), which can help diagnose PKS [9–11].

## Results

All ten patients had PKS confirmed by a fibroblast or amniotic fluid karyotype or by buccal smear MLPA. The presence of i(12)(p10) in the fibroblast karyotype ranged from 50 % to 100 % and in amniotic fluid from 75 % to 100 % (Table 1).

**Table 1:** Results of karyotype, MLPA (according to ISCN, 2013) and age at diagnosis

Cases	Results	% cells with i12p	Tissue	Age at diagnosis
1	47,XX,+i(12)(p10)/46,XX	82	Skin	3mo 24 d
2	47,XX,+i(12)(p10)/46,XX	50	Skin	10mo
3	47,XX,+i(12)(p10)/46,XX	80	Skin	1y10mo
4	47,XX,+i(12)(p10)	100	Skin	1y5mo
6	47,XX,+i(12)(p10)/46,XX	75	Amniotic fluid	Prenatal
	47,XX,+i(12)(p10)	100	Skin	2y 4 mo
	MLPA rsa 12p13.3(RBBP2)X4 and rsa 12p13.3(SLC6A12)X4	NA	Buccal smear	2y 4 mo
	MLPA rsa 12p13.3(RBBP2)X4 and rsa 12p13.3(SLC6A12)X4	NA	Skin	2y 4 mo
7	47,XY,+i(12)(p10)/46, XY	87	Skin	7 mo
8	47,XY,+i(12)(p10)	100	Skin	3 y
9	47,XY,+i(12)(p10)	100	Amniotic fluid	Prenatal
10	MLPA rsa 12p13.3(RBBP2)X4 and rsa 12p13.3(SLC6A12)X4	NA	Buccal Smear	1 y 10 mo

NA - not applicable.

Peripheral blood karyotypes were normal in nine patients. In one who died at 5 days of age it was not performed but diagnosis was confirmed by amniotic fluid karyotype.

**Fig. 1:**
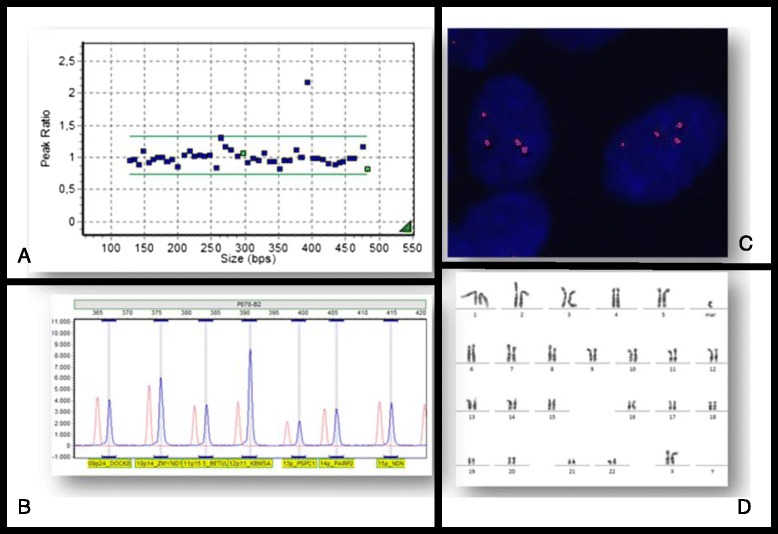
**a** and **b**: Histogram showing MLPA analysis of DNA from buccal smear; **c**: FISH in interphase cells showing 12p specific subtelomeric probe (Aquarius®, Cytocell Cambridge, UK ) hybridization; **d**: Fibroblast skin karyotype showing isochromosome 12p in G band

Three patients, with clinical features of PKS, had buccal smear MLPA confirming the diagnosis. In two patients fibroblasts culture failed and MLPA was performed in buccal smear sample showing four copies of the short arm of chromosome 12 (Fig. 1). In another patient, who had fibroblast karyotype, it was also performed MLPA of buccal smear and fibroblast in order to confirm that MLPA was able to diagnosis PKS.

**Fig. 2:**
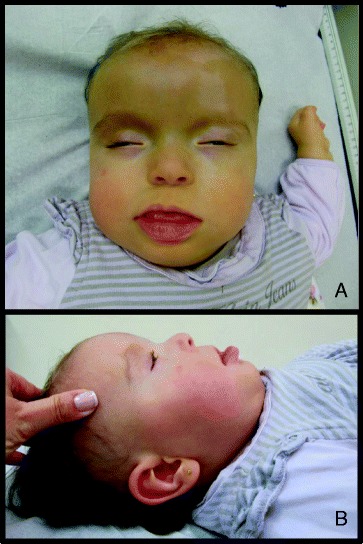
Patient # 5: dysmorphic features described in PKS: frontotemporal alopecia, hypertelorism, anteverted nostrils, long philtrum with “Pallister lip”, hypopigmented streaks. **a**: frontal view; **b**: lateral view

Clinical aspects are shown in Table 2. All patients presented clinical features associated with PKS (Fig. 2). Two of these patients were previously described [12]. Age at diagnosis varied from the prenatal period to 1y10mo.

**Table 2:** Clinical features

Case	Gender	Maternal/Paternal age at conception	Craniofacial	Dermatological	Gastroin-testinal/Genitourinary	Musculoskeletal	Cardiac	Otologic/Auditory	Ophthalmologic	CNS	Brain image
1	Female	18y/22y	Flat, broad nasal root, upslanting palpebral fissures, hypertelorism, coarse face, anteverted nostrils, retrognathia, small ears	Hypopigmented streaks	Anteriorly placed anus, Umbilical hernia	Normal	Patent ductus arteriosus	NA	Normal	Developmental delay	Periventricular leukomalacia
2	Female	29y/32y	Frontotemporal alopecia, long philtrum, hypertelorism, thin upper lip, anteverted nostrils, sparse eyebrows	Normal	Normal	Normal	Normal	Unilateral hearing loss	Normal	Seizures and developmental delay	Callosum disgenesy, shortage white matter, rarefied myelinization, kyphosis
3	Male	36y /43y	Hypertelorism, flat, broad nasal root, anteverted nostrils, thin upper lip, high-arched palate	Normal	Inguinal and umbilical hernia	Normal	Atrial septal defect (aneurysm of interatrial septum)	NA	NA	Hypotonia	Normal cranial CT
4	Female	36y/37y	Epicanthus, retromicrogna-thia, coarse face, hypertelorism, anteverted nostrils, thin upper lip, frontotempo-ral alopecia	Hypopigmented and hyperpigmen-ted streaks	Normal	Normal	Normal	Otitis and bilateral hearing loss	Hypermetropia and nystagmus	Developmen-tal delay, hypotonia	Normal MRI
5	Female	37y/NA	Hypertelorism, blepharophi-mosis, convex philtrum, high-arched palate, sparse anterior scalp hair	Hypopigmented streaks	Normal	Normal	Atrial septal defect	Bilateral hearing loss	Strabismus, diffuse abnormality of the retinal pigment epithelium	Developmental delay	Enlargement of the arachnoid space and meningeal artery tortuous
6	Female	34y/33y	Hypertelorism, long phlitrum, “Pallister-lip”, upslanting palpebral fissures, sparse eyebrows, macroglossia, bifid uvula	Hypopigmented streaks	Normal	Normal	Patent ductus arterious, atrial septal defect, pulmonary hypertension	NA	NA	Developmental delay and hypotonia	Thin corpus callosum
7	Male	39y/41y	Frontotempo-ral alopecia, hypertelorism, proeminent forehead, long philtrum, sparse eyebrow, flat, broad nasal root, anteverted nostrils, thin upper lip, “Pallister-lip”	Hyperpigmented streaks	Normal	Normal	Atrial septal defect	Unilateral sensorineural hearing loss	Thinning of retinal epithelium	Hypotonia and seizures	Normal cranial CT
8	Male	31y/NA	Upslanting palpebral fissures, ptosis, epicanthal folds, flat, broad nasal root , anteverted nostrils, retromicrogna-thia, long philtrum, frontotemporal alopecia, sparse anterior scalp hair	Normal	Umbilical hernia	Bilateral hip dislocation	Atrial septal defect (aneurysm of interatrial septum)	NA	NA	Hypotonia and seizures	NA
9	Male	16y/NA	Hypertelorism, broad nasal root, cleft lip and palate,	Normal	Imperforate anus, renal agensis - right side, cryptorchidism	Postaxial polydactyly in hands and foot	Ventricular septal defect, Double Outlet Right Ventricle	NA	NA	Hypotonia	Cranial Ultrasound: periventricular hyperechogenic cyst
10	Female	23y/25y	Upslanting palpebral fissures, bitemporal alopecia, “Pallister-lip”, anteverted nostrils, broad nasak root, long philtrum	Hypopigmented and hyperpigmented streaks	Normal	Normal	Normal	NA	Strabismus	Developmen-tal delay, hypotonia	Cranial ultrasound: normal

NA - not applicable.

Two patients died: one at 5 days due to respiratory problems and other at 14 years old due to pneumonia. Two patients have lost follow-up.

Current age of six patients ranges from 1y9mo to 19 years.

## Discussion

Although typical clinical features of PKS commonly exist, clinicians often do not raise the possibility of this diagnosis. Another factor that makes this diagnosis difficult is the need for cytogenetic analysis of tissues other than peripheral blood.

Even though there is no pathognomonic feature of PKS, there are some typical findings such as coarse facies, prominent forehead, bitemporal alopecia, upslanting palpebral fissures, cleft palate, sparse eyebrows, pigmentary skin anomalies including hyperpigmented and hypopigmented streaks, developmental delay, hypotonia and seizures. It is important to note that some of these findings might not be evident at birth; the coarsening of the face occurs as the child ages [2].

All patients described in our study exhibited typical clinical features of PKS, and parental age at conception was in accordance with that reported in the literature [3].

Cardiac malformations are common in PKS patients described in the literature; the most common malformations are patent foramen ovale, atrial septal defects and patent ductus arteriosus [3, 13]. Two of our patients had aneurysm of the interatrial septum, which had not been previously described in PKS.

Skin anomalies, such as hyperpigmented and hypopigmented streaks, were observed in 6/10 patients. Despite being a frequent finding in PKS patients, such skin anomalies are considered suggestive of PKS and not a mandatory feature.

With regard to the ophthalmological findings, one patient had a diffuse abnormality of retina pigmentation, which has been previously described by Graham et al. This may suggest a retinal pigment mosaicism similar to that described in skin [14].

Gastrointestinal malformations were found less frequently in our cohort than described in the literature, although the malformations were similar to those previously described: displacement of the anus and umbilical hernia. Diaphragmatic hernias, present in 11–29 % of reported cases, were not observed in our patients [3].

Hearing and ophthalmologic impairment were observed less frequently in our population compared to reports in the literature. Only one patient had bilateral hip dislocation.

Compared to the literature, seizures were observed less frequently in our cohort. Because the onset of seizures in PKS patients might occur between the ages of 2 to 5 years, some of our patients might develop seizures later in life. There is no typical seizure pattern in PKS patients [15–17].

Similar to our findings, karyotypes of peripheral blood are usually normal. Wenger et al. showed that only 3 % of cells from peripheral blood exhibited i(12)(p10), whereas analysis of skin and lung tissues that were obtained postmortem exhibited i(12)(p10) in 98.5 % and 97.5 % of the cells, respectively [9]. Liehr et al. described a variety of PKS’s karyotypes: complete trisomy 12 in mosaicism: 47,XX,+12[20 %]/47,XX,+i(12)(p10)[80 %], hexasomy 12p: 48,XX,+i(12)(p10),+i(12)(p10)[16 %], even when PKS phenotype is typical [18]. More studies are necessary to stablish if there is a clinical correlation with the cytogenetics findings.

We used MLPA method for diagnosis with DNA prepared from buccal smear samples in three patients, with the detection of four copies of the short arm of chromosome 12, confirming the diagnosis of PKS. There are few reports of PKS diagnosis using MLPA and DNA from buccal smears in the literature. We recommend this analysis as the first exam, before a skin biopsy.

MLPA assay is a recently developed technique that is able to detect variations in the genes copy number and dosage. This test is a high throughput analysis, allowing up to 96 samples to be handled simultaneously allowing the study of several regions of the human genome in a single reaction [19, 20].

Due to this capability, MLPA can be applied in the molecular diagnosis of several syndromes since the duplication of entire genomic regions causes a disease due to the presence of extra copies of the genes, while complete or partial deletion can produce a completely different phenotypic effect [19].

In fact, MLPA assay is one of the most widely used techniques for the molecular investigation of microdeletion/microduplication syndromes. The use of this technique has important advantages such as relative simplicity of approach, low cost, rapid turnaround, ease of multiplexing to permit high confidence in the results, high accuracy of copy number estimation, and the potential for combination of copy number analysis with other applications, such as methylation detection or SNP genotyping [19, 20].

MLPA use target sequences, between 50–80 nucleotides, that applied to DNA extracted from a buccal swab allow identifying genomic aberrations, easier than detected by FISH. Over 300 kits are so far commercially available, dedicated to the study of several diseases [19].

Finally compared to array CGH (Comparative Genomic Hybridization) and FISH (Fluorescent *In situ* Hybridization) whose have been previously described as alternatives to diagnose PKS, MLPA technique is less expensive and technically uncomplicated method [19].

MLPA on buccal smears is also non-invasive and yields a shorter turnaround time for results compared to a fibroblast karyotype. A limitation of MLPA is that mosaicism in low level may not be detected, so a normal MLPA result cannot rule out PKS diagnosis [20].

## Conclusion

A precocious PKS diagnosis is important to optimally manage the disease and to provide genetic counseling.

MLPA on buccal smears is a good and non-invasive method to detect extra copies of 12p and should be considered as the first exam, before a skin biopsy for a fibroblast karyotype is performed.

Thus we highlight the importance of associate cytogenomic approaches in clinical genetics, to provide additional information relevant to confirm the diagnosis, for patient management and genetic counseling.

### Methods and patients

This is a descriptive study based on the medical records of ten patients with a confirmed diagnosis of PKS who were followed in the Genetics Unit of the Instituto da Criança (Brazil) from 1990 to 2014. The study was approved by the by the ethics committee of the HCFMUSP (University of São Paulo-CAPPesq) and informed consent form was obtained from all families.

### Cytogenomic analysis

Peripheral blood karyotype was performed using a standard phytohemagglutinin-stimulated lymphocyte culture method followed by G-banding. Twenty metaphase cells were analyzed for all patients except one, who died at 5 days of age. Also, we performed a skin biopsy for fibroblast karyotype and the long-term closed flask fibroblast explant cultures were set up according to the protocol adapted from Rooney [21]. Additional Fluorescence *in situ* hybridization (FISH) with chromosome 12p specific subtelomeric probe (Aquarius®, Cytocell Cambridge, UK) according to the technique of Pinkel [22], with minor modifications was carried out on interphase nuclei of subcultured fibroblasts.

In three of the patients buccal smear was collected with the Oragene Kit (Ottawa, Canada) and DNA was extracted using the QIAamp DNA Blood Midi Kit 250 (QIAGEN, Valencia, California) in order to perform Multiplex Ligation-dependent Probe Amplification (MLPA-MRC Holland, Amsterdam, The Netherlands) assay.

MLPA with kits SALSA P070 and SALSA P036 that contain subtelomeric probes, *RBBP2* and *SLC6A12* respectively, determine the DNA copy number variations in 12p region and can provide a definitive diagnosis.

## Consent

Written informed consent was obtained from the parents of the patients.
